# List of Diseases in October, in the Practice of Dr. Fothergill

**Published:** 1813-11

**Authors:** 


### *List of Diseases in October, in the Practice of* DR. FOTHERGILL.

| | |
|---|---|
| RHEUMATISMUS | 10 |
| Febris | 2 |
| Scarlatina Anginosa | 3 |
| Scarlatina Maligna | 2 |
| Cynanche Tonsillaris | 2 |
| Cynanche Laryngea | 1 |
| Phrenitis | 2 |
| Peripneumonia | 1 |
| Pertussis | 4 |
| Tussis et Dyspnœa | 12 |
| Catarrhus | 8 |
| Phthisis Pulmonalis | 5 |
| Marasmus | 2 |
| Hæmoptoe | 1 |
| Hæmatemesis | 2 |
| Asthenia | 7 |
| Cephalalgia | 3 |
| Lumbago | 3 |
| Colica | 2 |
| Gastrodynia | 4 |
| Vomitus | 2 |
| Dyspepsia | 6 |
| Diarrhœa | 3 |
| Dysure | 2 |
| Erythema Nodosum | 1 |
| Erysipelas | 1 |
| Lichen Simplex | 1 |
| Porrigo | 2 |
| Menorrhagia | 2 |
| Amenorrhœa | 4 |
| Morbi Infantiles | 3 |

